# Supplementary material for: Identification and immunoinfiltration analysis of key genes in ulcerative colitis using WGCNA
Source: PeerJ. 2024 Feb 26;12:e16921. doi: 10.7717/peerj.16921 (PMC10903335; doi:10.7717/peerj.16921)
Supplement: Supplemental Information 1 [file peerj-12-16921-s001.zip › Supplementary Figures/Supplementary Figure 1/Supplementary Figure 1.pdf]

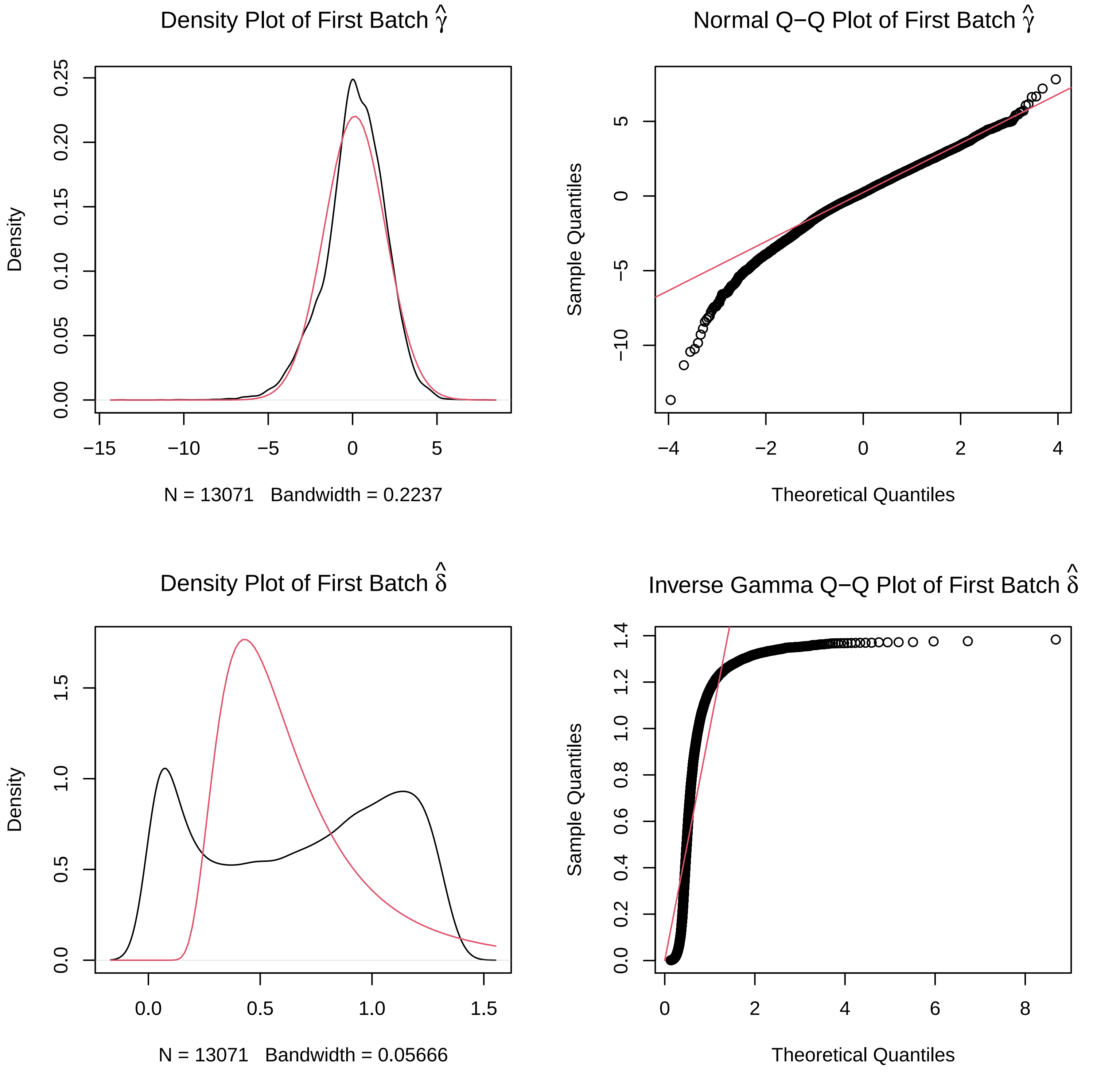

Density Plot of First Batch $\hat{\gamma}$
Density
0.00
0.05
0.10
0.15
0.20
0.25
−15
−10
−5
0
5
N = 13071 Bandwidth = 0.2237
Normal Q−Q Plot of First Batch $\hat{\gamma}$
Sample Quantiles
−10
−5
0
5
−4
−2
0
2
4
Theoretical Quantiles
Density Plot of First Batch $\hat{\delta}$
Density
0.0
0.5
1.0
1.5
0.0
0.5
1.0
1.5
N = 13071 Bandwidth = 0.05666
Inverse Gamma Q−Q Plot of First Batch $\hat{\delta}$
Sample Quantiles
0.0
0.2
0.4
0.6
0.8
1.0
1.2
1.4
0
2
4
6
8
Theoretical Quantiles
