# Supplementary figures and images for: Identification and immunoinfiltration analysis of key genes in ulcerative colitis using WGCNA
Source: PeerJ. 2024 Feb 26;12:e16921. doi: 10.7717/peerj.16921 (PMC10903335; doi:10.7717/peerj.16921)

PCA after of batch

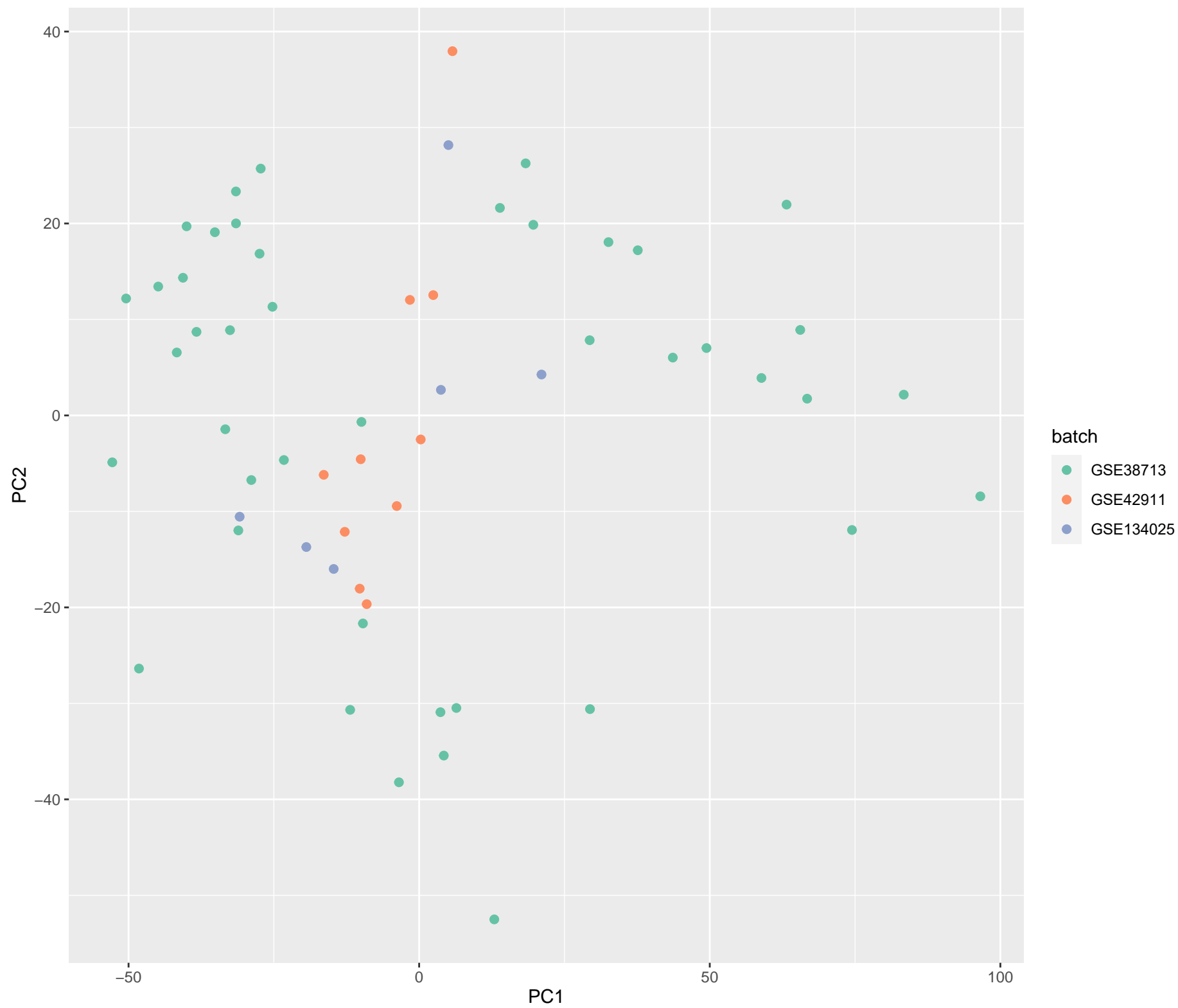

Supplement: Supplemental Information 1 [file peerj-12-16921-s001.zip › Supplementary Figures/Supplementary Figure 2/PCA-after-batch.pdf]

PCA after of State

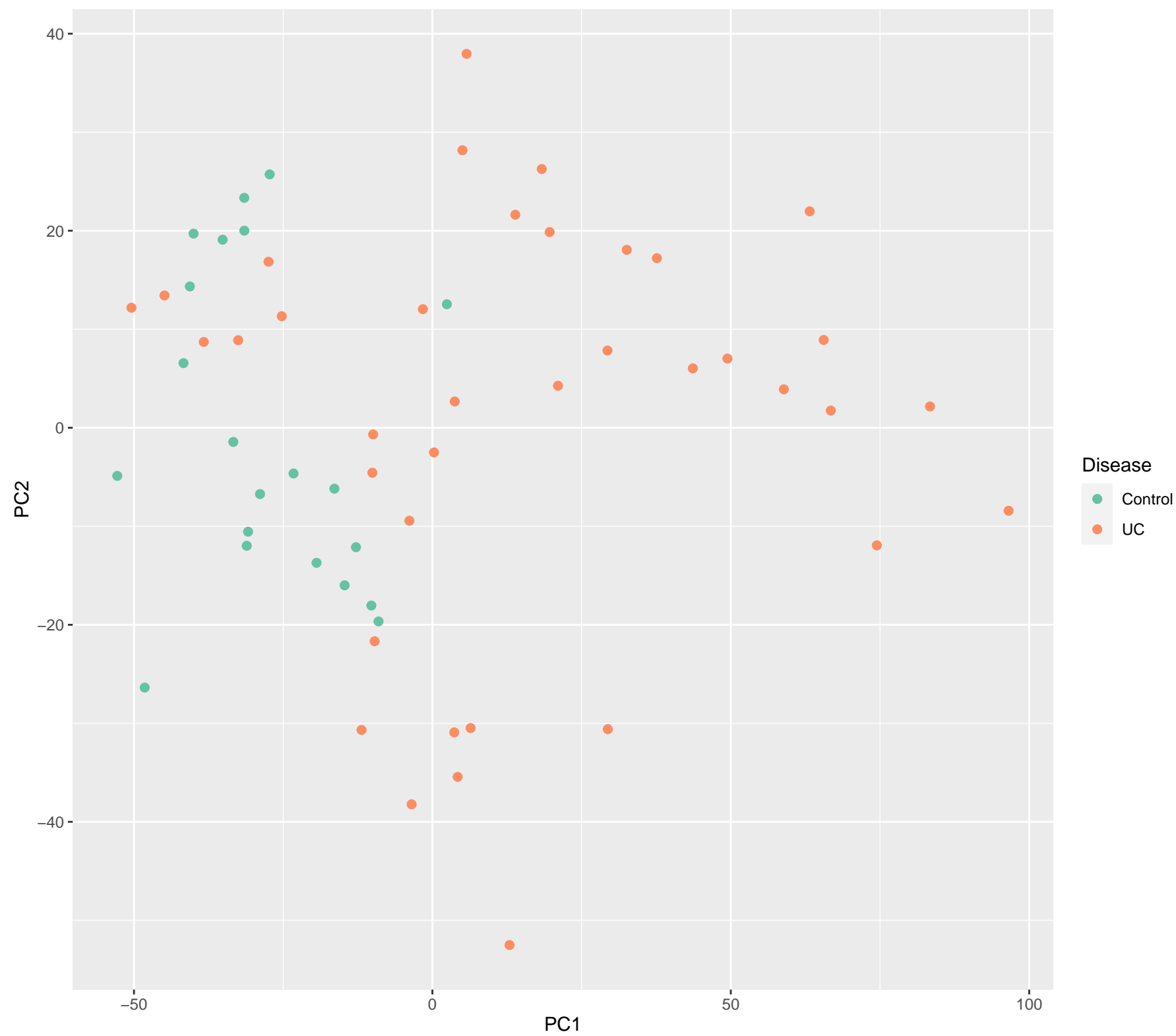

Supplement: Supplemental Information 1 [file peerj-12-16921-s001.zip › Supplementary Figures/Supplementary Figure 2/PCA-after-state.pdf]

PCA before of batch

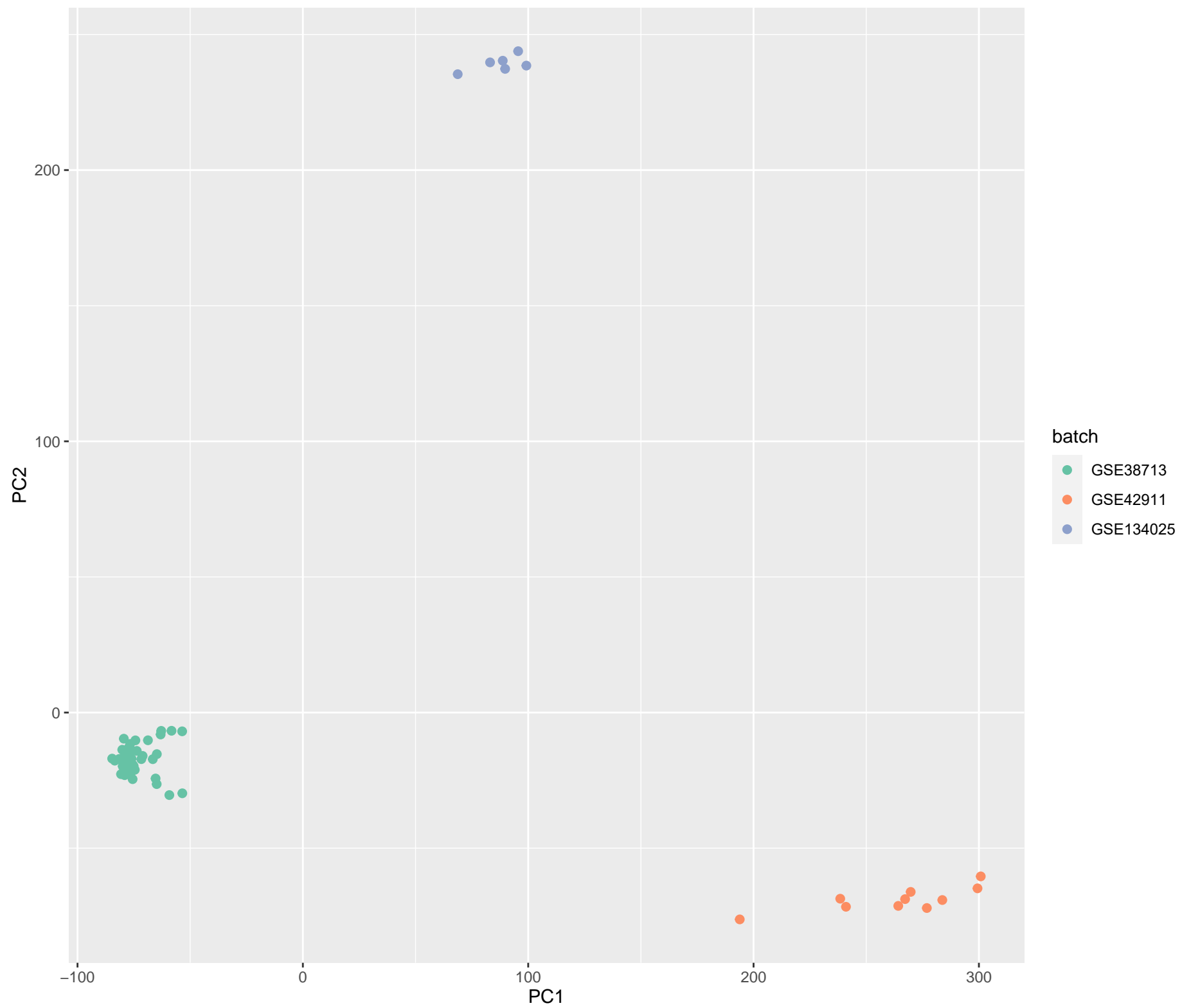

Supplement: Supplemental Information 1 [file peerj-12-16921-s001.zip › Supplementary Figures/Supplementary Figure 2/PCA-before-batch.pdf]

PCA before of State

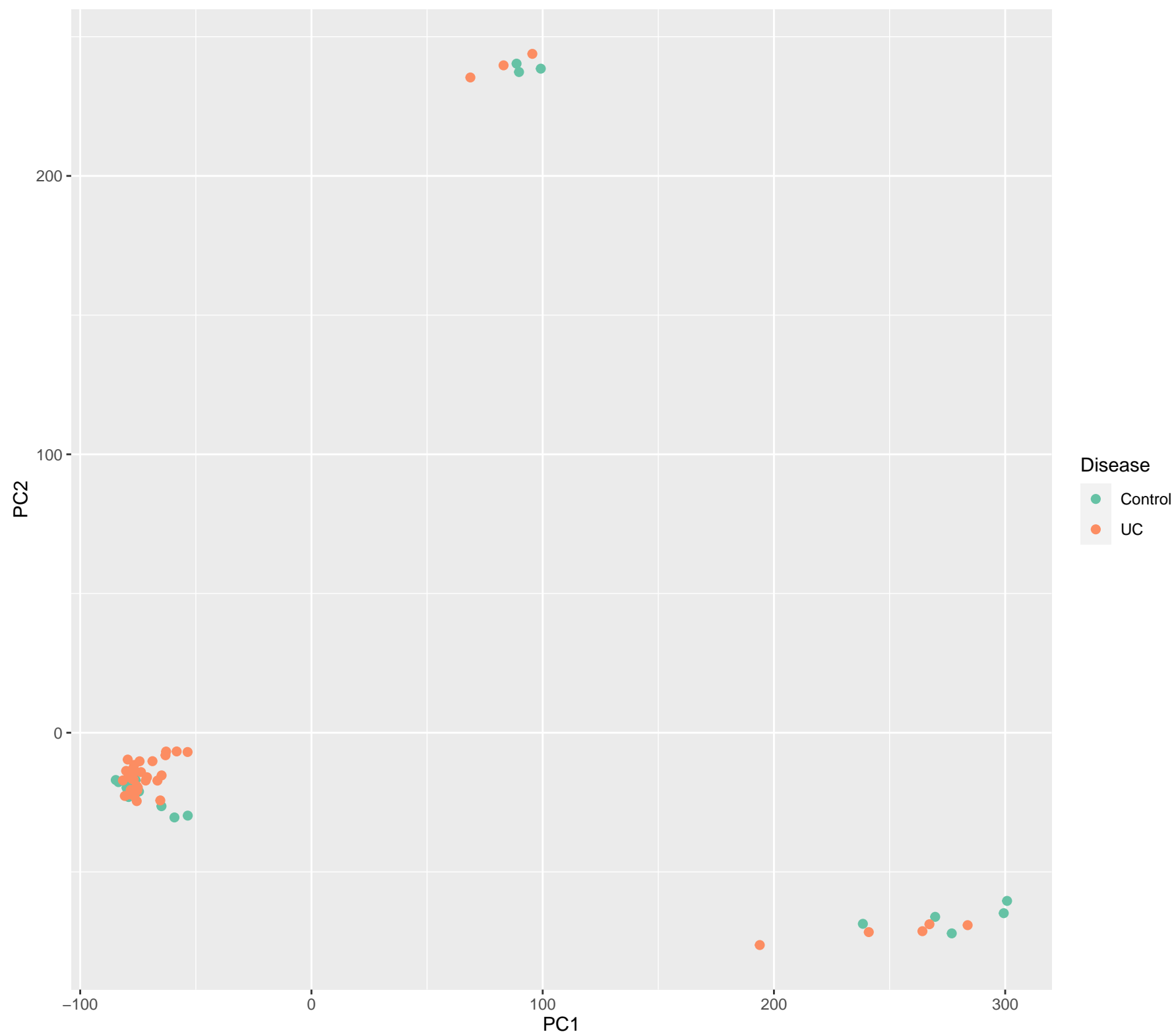

Supplement: Supplemental Information 1 [file peerj-12-16921-s001.zip › Supplementary Figures/Supplementary Figure 2/PCA-before-state.pdf]
